# Is type 2 diabetes associated dementia a microvascular early-Alzheimer’s phenotype induced by aberrations in the peripheral metabolism of lipoprotein-amyloid?

**DOI:** 10.3389/fendo.2023.1127481

**Published:** 2023-02-16

**Authors:** Ryusuke Takechi, Arazu Sharif, Emily Brook, Maimuna Majimbi, Dick C. Chan, Virginie Lam, Gerald F. Watts, John C. L. Mamo

**Affiliations:** ^1^ Curtin Health Innovation Research Institute, Curtin University, Perth, WA, Australia; ^2^ Departments of Cardiology and Internal Medicine, Royal Perth Hospital, School of Medicine, University of Western Australia, Perth, WA, Australia

**Keywords:** amyloid, diabetes, Alzheimer’s, lipoprotein, triglycerides

## Abstract

There is increasing evidence of a positive association of type 2 diabetes with Alzheimer’s disease (AD), the most prevalent form of dementia. Suggested pathways include cerebral vascular dysfunction; central insulin resistance, or exaggerated brain abundance of potentially cytotoxic amyloid-β (Aβ), a hallmark feature of AD. However, contemporary studies find that Aβ is secreted in the periphery by lipogenic organs and secreted as nascent triglyceride-rich lipoproteins (TRL’s). Pre-clinical models show that exaggerated abundance in blood of TRL-Aβ compromises blood-brain barrier (BBB) integrity, resulting in extravasation of the TRL-Aβ moiety to brain parenchyme, neurovascular inflammation and neuronal degeneration concomitant with cognitive decline. Inhibiting secretion of TRL-Aβ by peripheral lipogenic organs attenuates the early-AD phenotype indicated in animal models, consistent with causality. Poorly controlled type 2 diabetes commonly features hypertriglyceridemia because of exaggerated TRL secretion and reduced rates of catabolism. Alzheimer’s in diabetes may therefore be a consequence of heightened abundance in blood of lipoprotein-Aβ and accelerated breakdown of the BBB. This review reconciles the prevailing dogma of amyloid associated cytotoxicity as a primary risk factor in late-onset AD, with substantial evidence of a microvascular axis for dementia-in-diabetes. Consideration of potentially relevant pharmacotherapies to treat insulin resistance, dyslipidaemia and by extension plasma amyloidemia in type 2 diabetes are discussed.

## 1 Introduction

Alzheimer’s disease (AD) is a progressive, neurodegenerative disorder characterised by gradual cognitive decline, deterioration of living activities and behavioural disturbances (1). AD is associated with hallmark neuropathologies, including extracellular deposition of amyloid-β (Aβ) peptide, intra-neuronal neurofibrillary tangles and synaptic degeneration (2). Numerous studies have reported an association between cerebral Aβ burden with decline in cognitive performance and memory (3, 4), so it is unsurprising that Aβ is considered an important therapeutic target to treat AD (5).

There is accumulating evidence that diabetes significantly increases risk for AD (1, 6). However, population studies do not consistently find greater rates of cerebral amyloid deposition in cohorts of people with diabetes per se (7). This paradox may reflect the pathological criteria which is used to clinically define AD, namely, requiring evidence of late-stage protein aggregate formation within brain parenchyme. However, in AD, microvascular disturbances precede frank amyloidosis decades before cognitive dysfunction, suggesting the alternate hypothesis and increasingly held view of a vascular origin for AD (8, 9). Cerebral capillary dysfunction is manifest in diabetes (10, 11), so it is a reasonable proposition that heightened risk for AD may be a consequence of heightened central microvascular sequalae.

In this review, we put forward a reconciliatory hypothesis that AD-in-diabetes may principally be a consequence of microvascular aberrations induced by exaggerated exposure to Aβ in blood, the latter, specifically a consequence of disturbances in triglyceride rich lipoproteins (TRLs). There is an increasing body of evidence to suggest that in type 2 diabetes (T2DM), greater vascular exposure to plasma lipoprotein associated Aβ compromises cerebral capillary integrity (1). Heightened extravasation of the lipoprotein-Aβ in T2DM amplifies neurovascular inflammation and neurodegeneration, resulting in accelerated neuronal cell death and premature cognitive decline. The indicated lipoprotein-Aβ microvascular axis pathway for AD in diabetes, we contend may be a future target for reducing risk for AD in diabetes.

## 2 Cerebral capillary integrity in Alzheimer’s disease

A unique feature of cerebral capillary vessels is the blood-brain barrier (BBB), describing apposed endothelial cells with tight junction proteins, basement membranes, astrocytes and pericytes that collectively restrict and regulate the diffusion of large, or hydrophilic molecules into cerebrospinal fluid (12–14). Increased capillary permeability is the first physiological aberration that defines BBB dysfunction, resulting in abnormal protein and macromolecular kinetics from blood-to-brain and endothelial cells may show decreased endothelial mitochondrial density and increased pinocytotic activity (15). The reduction of endothelial tight junction proteins and extravasation of plasma molecules in AD brains stimulates functional changes in astrocytes, which develop a pro-inflammatory phenotype (16). Chronic BBB disturbance will ordinarily lead to exaggerated deposition of extracellular proteoglycans and collagen, creating a cycle of plasma derived proteins, macromolecular and cellular deposition, inflammation, a reduction in distensibility and convolutional abnormalities of the surrounding neurons (17).

The breakdown of cerebral capillary BBB is reported in late onset AD and indeed ordinarily precedes by decades the pathophysiological hallmarks of amyloidosis, tau hyperphosphorylation, cognitive decline and brain atrophy, consistent with causality (12). Commonly reported along with the BBB breakdown is the cerebral amyloid angiopathy, which describes the abnormal deposition of Aβ within the cerebral microvessels.

In summary, the above observations suggest that capillary BBB dysfunction may be pivotal to the aetiological mechanisms of cognitive decline in AD.

## 3 Peripheral metabolism of amyloid, cerebral capillary integrity and neurovascular inflammation

Aβ is a normal soluble component of blood and recent studies indicate that the relative abundance of the pro-amyloidogenic Aβ_1-42_ isoform relative to Aβ_1-40_, can identify ~90% of subjects who progress to clinical AD (18). Evidence of a causal association with risk for AD comes from pre-clinical findings. Studies in primates and guinea pigs showed that intravenous infusion of radiolabelled Aβ resulted in substantial blood-to-brain influx concomitant with heightened inflammation of the neurovascular unit (19). Contemporary evidence was indicated in peripheral vascular parabiotic studies of wild-type mice and human-amyloid transgenic mice (20). In this study, the researchers surgically fused blood vessels of a wild-type mouse with transgenic amyloid mouse. The wild-type mice developed cerebral amyloid plaques and tau hyperphosphorylation within 4 months of surgical intervention. The parabiotic wild-type mouse had accelerated neurodegeneration, neuroinflammation and microhemorrhages, however, capillary integrity was unfortunately not reported.

Aβ is highly lipophilic and Matsubara et al. showed that ~90% of Aβ_1-40_ and ~97% of Aβ_1-42_ in the circulation are bound to lipoproteins (21), particularly TRLs including postprandial chylomicrons and hepatically-derived very-low-density lipoproteins (VLDL). Kinetic studies suggest that the metabolism of TRL-Aβ follows the catabolic fate of the native lipoprotein remaining with the TRL until normally cleared by liver (22). Indeed, Aβ probably serves as an apolipoprotein, regulating TRL metabolism. Several studies have demonstrated that brain parenchymal extravasation of lipoprotein-Aβ may significantly exacerbate cerebral amyloid load in AD, particularly when capillary integrity is compromised and barrier function is reduced, amplifying inflammation of the neurovascular unit (22–24).

The hypothesis of a lipoprotein-Aβ capillary axis for AD was directly considered in a recent study by Lam et al. (25). Mice were genetically engineered to produce human Aβ exclusively in liver, not CNS. Lam et al. reported blood-brain barrier dysfunction, neurovascular inflammation, accelerated evolution of age-associated lipid inclusion bodies in brain parenchyme, concomitant with increased brain parenchymal abundance of Aβ, early onset neurodegeneration and brain atrophy. Lam et al. also reported that the mice performed poorly on hippocampal dependent learning challenge, consistent with causality. The vascular phenotype demonstrated in the Lam et al. study is consistent with the accumulating body of evidence supporting the hypothesis of a microvascular axis trigger for AD.

**Figure 1:**
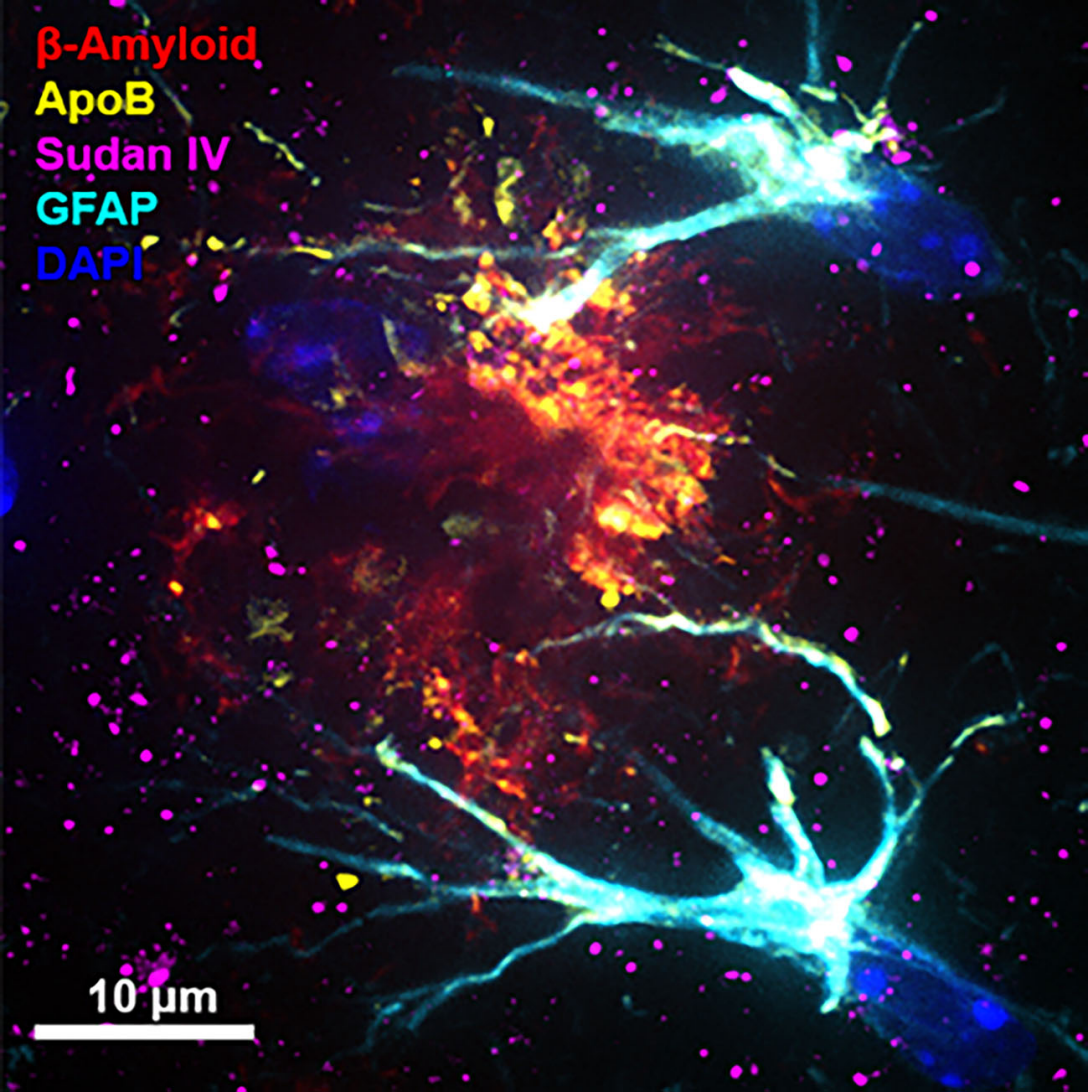
Immunomicrograph of amyloid plaque colocalisation. The immunofluorescent microscopy image shows an amyloid plaque in one of the most commonly used Alzheimer’s model, double transgenic human amyloid precursor protein-presenelin 1 (APP/PS1) mice. Cerebral immunofluorescent microscopy image was captured in 12-month old APP/PS1 mouse brain. Amyloid plaques, triglyceride rich lipoproteins, neutral lipids and activated astrocytes were detected with anti-amyloid-β (red), anti-apolipoprotein B (apoB) (yellow), Sudan IV (magenta), and glial fibrillary acidic protein (GFAP) (cyan), respectively. Nuclei was counterstained with DAPI (blue) and the scale bars indicate 10 µm. (Original image from the authors generated for this article. For detailed methods, please refer to (25).

Several other lines of evidence are consistent with the lipoprotein-Aβ capillary risk axis for AD. In wild-type mice, a well-tolerated saturated-fat enriched diet was found to strongly stimulate biosynthesis of nascent TRL-Aβ, concomitant with a reduction in capillary endothelial tight junction proteins; marked blood-to-brain extravasation of plasma-derived proteins and macromolecules including TRL-Aβ and astroglial cell activation (23, 26–28). In other studies, Burgess et al. also reported that in some human amyloid transgenic mice with ubiquitous expression of human amyloid, onset and progression of cerebral amyloidosis was strongly associated with secretion into blood of TRL-Aβ (29). Herein, we also show in transgenic amyloid mice remarkable colocation of apolipoprotein B (a marker of nascent hepatic and intestinal lipoproteins) with amyloid plaque and the accumulation of neutral lipids (**Figure 1**).

In summary, these data collectively suggest that perturbation of peripheral TRL-Aβ metabolism may increase the blood-to-brain transport of Aβ through disruption of the BBB, significantly contributing to the onset and progression of AD.

## 4 Triglyceride-rich lipoprotein amyloid metabolism in diabetes

Dyslipidemia and alterations in lipoprotein metabolism in T2DM have been extensively studied and reviewed (30). Collectively, the body of literature suggests that aberrations in lipid and glucose metabolism in T2DM are particularly relevant in the context of macrovascular and microvascular disease, respectively (31). However, there are a paucity of studies investigating whether lipoprotein-Aβ metabolism modulates cerebral capillary function *per se* in T2DM.

The prevalence of dyslipidemia in T2DM is significant (>50%) and ordinarily mixed, showing increased plasma abundance of intestinal and hepatic derived TRL, which is as a consequence of decreased hydrolysis of TRL-triglyceride, decreased shedding of excess lipoprotein phospholipids to generate nascent high-density lipoprotein (30). Subjects with T2DM are not ordinarily hypercholesterolemic, however there is preponderance of smaller-dense and more atherogenic and triglyceride enriched LDL compared with subjects without diabetes. Diabetic dyslipidaemia is primarily due to hepatic insulin resistance and post secretion from lipogenic organs, aberrations in catabolism. Insulin resistance in T2DM, rather than hyperinsulinemia results in overproduction of TRL (31). Insulin stimulates vascular endothelial expression of lipoprotein lipase, which converts TRL to the high receptor-uptake remnant isoform and insulin also increases expression of the key receptor required for lipoprotein clearance, the apo B/E receptor (LDL-receptor) (32).

**Figure 2:**
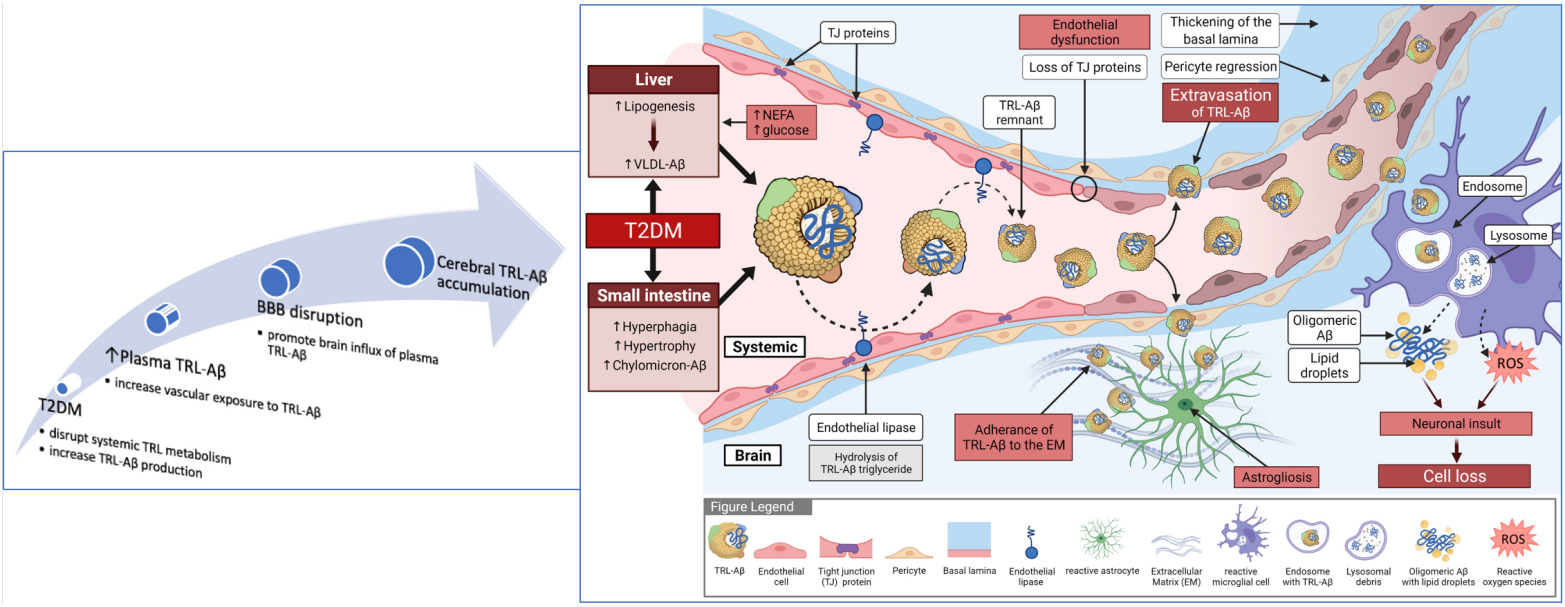
Summary of blood to brain lipoprotein-Aβ hypothesis for diabetes-induced Alzheimer’s disease. Exaggerated provision of substrate from plasma (non-esterified fatty acids and glucose) and hyperphagia drives lipogenesis. Loss of insulin sensitivity results in exaggerated synthesis and secretion of nascent triglyceride-rich very-low-density lipoproteins from liver and chylomicrons from the small intestine. Amyloid-beta is an apoprotein of TRL, however with exaggerated vascular exposure to TRL-Aβ because of overproduction and decreased lipolysis, expression of endothelial tight-junction proteins is compromised and capillary permeability increased. Extravasation of lipoprotein-Aβ within brain parenchyme triggers astrogliosis. Uptake of lipoprotein-Aβ by activated glial cells can lead to secretion of free amyloid and greater propensity for oligomerisation and fibrillar formation. In addition, the mitochondrial respiratory burst in glial cells will result in synthesis and secretion of highly reactive oxygen species that may further compromise the neurovascular unit, including neuronal death.

The disturbances in TRL metabolism seen in insulin resistant/T2DM may result in exaggerated vascular exposure to TRL-Aβ and micro-angiopathy, increasing the risk for earlier onset Alzheimer’s (**Figure 2**). A direct effect of insulin, or insulin-like growth factor on TRL-Aβ metabolism may also occur. Based on high-fat feeding murine models, it is a reasonable proposition that insulin resistance concomitant with exaggerated abundance of lipogenic substrate in T2DM (glucose, hyperphagia and intestinal hypertrophy) could stimulate synthesis and secretion of TRL-Aβ (28, 33, 34). Studies in our laboratory in a dietary-induced murine model of diabetes are consistent with exaggerated production of TRL-Aβ from the intestine (9, 27). In other contemporary studies exploring the association between T2DM and AD, insulin/IGF-1 signaling had significant downstream effects on gene expression (35). In cell culture studies of neuronal cells, insulin suppressed phosphorylation of amyloid precursor protein and in a fat-fed murine model of metabolic syndrome, induction of insulin resistance abolished the inhibitory effect of insulin (36). By extension, similar effects may be realised in peripheral tissues and organs, resulting as a consequence of insulin resistance, in increased genesis of Aβ in hepatocytes and enterocytes secreted with nascent TRL. Other pathways responsible for heightened plasma TRL-Aβ in T2DM may also be a consequence of decreased degradation. Clinical studies showed in otherwise healthy subjects that insulin was positively associated with increased catabolism of plasma Aβ (37), raising the possibility that in insulin resistant T2DM, the degradation pathway is impaired. Consistent with the latter and increased risk for AD in T2DM, Ekblad et al. investigated whether midlife insulin resistance is an independent risk factor for brain amyloid accumulation (38). They reported a two-fold increase in cerebral amyloidosis detected by PET scanning in the insulin resistant group associated with higher midlife HOMA-IR scoring and independent of apo E genotype. Moreover, in recent studies, Banks et al. showed that human CSF contains triglyceride and that peripheral blood radiolabelled triolein readily crossed the BBB (39). The latter induced leptin and/or insulin resistance at hypothalamic receptors and blocked satiety of centrally administered leptin.

In summary, the above data support the notion that insulin resistance in T2DM may significantly disturb plasma Aβ homeostasis *via* impaired metabolism of TRL-Aβ metabolism, which may explain the mechanisms by which T2DM increases the risk of accelerated cognitive decline and AD (**Figure 2**).

## 5 Apolipoprotein E genotype, lipoprotein metabolism, Alzheimer’s disease, and diabetes

Apolipoprotein E is a 34kDa protein synthesized principally in liver and in the context of peripheral lipoprotein metabolism, recognized for its pivotal role in serving as the binding ligand for receptor mediated clearance of triglyceride depleted remnant lipoproteins of chylomicrons and VLDL (31).

In humans, three primary isoforms are indicated with apo E2 generally favourable for cardiovascular and neurological health and apo E4 least favourable. In subjects heterozygous for APOE4/E3, apo E4 preferentially distributes to lipoproteins richer in TG which leads to downregulation of the apo B/E receptor (40). Apart from ageing, APOE4 is also the most significant established risk factor for late-onset AD, increasing the likelihood of disease by 17% and 43% for hetero- and homozygosity respectively and decreasing age of onset (41). Expressing APOE4 results in cognitive deficits being realised approximately 10 years earlier compared to subjects with APOE2 or APOE3 alleles (42). Remarkably, between 65-80% of AD patients carry APOE4 compared to 25% in the general population.

Reflecting the prevailing dogma that AD has a central aetiology, studies investigating potential mechanisms underpinning association of APOE4 genotype Alzheimer’s risk have focussed on effects on amyloid precursor processing, potential effects on oligomerisation and degradation, on synaptic plasticity, tau phosphorylation, neuroinflammation and modulating TREM-2 mediated microglial phagocytosis (42). However, despite being lipidated in biological fluids, there is a paucity of studies that have considered the effect of APOE4 genotype on plasma homeostasis of lipoprotein-Aβ and the potential microvascular sequalae. Based on the suppressive effects on clearance of TRL-remnants post triglyceride hydrolysis, notionally APOE4 may exacerbate accumulation of TRL-Aβ and accelerate degeneration of the microvasculature. Other contemporary considerations for synergistic effects of APOE genotype is regulation of hematopoiesis, blood monocyte activation, vascular inflammation and vascular tone (43). In line with our hypothesis for AD in diabetes, apoE4 genotype significantly increases the risk of diabetes (44) and exaggerates the dyslipidaemia further increasing the plasma levels of TRLs (45).

In summary, the apoE4 genotype may further perturb the metabolism of TRL-Aβ in T2DM amplifying microvascular corruption and increasing risk of AD in diabetes.

## 6 Targeting peripheral metabolism of lipoprotein-Aβ to reduce diabetes associated Alzheimer’s disease

Population studies show heightened risk of AD with diets enriched in saturated fatty acids and reduced risk with a Mediterranean diet, statin therapy and APOE ϵ2 genotype (46). Potential mechanisms for these associations could include a reduction in secretion of nascent TRL-Aβ *via* a Mediterranean diet, or statins and greater rates of clearance in subjects heterozygous for APOE2. Another cohort of patients’ worthy of considering TRL-Aβ metabolism and homeostasis is in the REDUCE-IT trial, a high-dose eicosapent-ethyl (4 g/day) intervention study in 8,179 statin-treated patients with triglycerides 136-500 mg/dL significantly reduced risk of ischemic events (47).

Probucol is a historic cholesterol-lowering drug that has been shown to have favourable effect on lipoprotein-Aβ in an experimental setting (48). In wild-type mice, probucol significantly markedly suppressed the enterocytic production of TRL-Aβ, normalizing the plasma levels of Aβ (48). Moreover, in diabetic mouse models, the provision of probucol resulted in the prevention of BBB disruption and neurocognitive decline (11). There is one study exploring the effects of probucol on cognition (ACTRN12621000726853), neurovascular integrity and plasma TRL-Aβ (49).

Newer generation lipid lowering strategies that may be relevant to the regulation of TRL-Aβ homeostasis and the integrity of the cerebral microcirculation. Inhibitors of proprotein convertase subtilisin-kexin type 9 (PCSK9) have demonstrated to lower the risk of cardiovascular disease, particularly in high-risk subjects such as those with T2DM. Potential effects of PCSK9 inhibitors on plasma amyloid homeostasis are not known, but to the contrary, on the basis that cognitive impairment in AD is associated with cholesterol metabolism, PCSK inhibitors are presently issued with a warning concerning possible negative impact on cognitive function. Interestingly, Picard et al. reported PCSK9 was elevated in frontal cortices of AD subjects compared to controls, both at the mRNA and protein levels (50). Zimetti et al. also reported higher PCSK9 in CSF of AD patients (51). Benn et al. undertook a Mendelian randomisation study to explore the hypothesis that genetic variants in genes PCSK9 *(and HMGCoA reductase*) controlling LDL-cholesterol metabolism were associated with AD, dementia and with Parkinson’s disease (52). Their conclusion was that low LDL-cholesterol due to PCSK9 or HMGCoA variants were not associated with increased risk for AD, so PCSK9 may be worthy of consideration in the context of T2DM. To that effect, in a high-fat fed rodent model where TRL-Aβ synthesis and secretion are expected to be exaggerated, PCSK9 inhibition and atorvastatin both reduced hippocampal apoptosis and amyloid protein synthesis (53). The PCSK9 treated group of rats showed greater amelioration of BBB-breakdown, microglial hyperactivity, hippocampal oxidative stress, synaptic dysplasticity and cognitive decline. The latter findings are consistent with the possibility that PCSK9 inhibitors potentially reduce risk for AD *via* a TRL-Aβ/capillary axis, however this remains to be considered.

Contemporary RNA-based antisense oligonucleotides and small interfering RNA’s for apoC3 and the angiopoietin-like 3 (ANGPTL3) profoundly attenuate plasma triglyceride in familial chylomicronemia, severe hypertriglyceridemia, dyslipidemia and T2DM (54). As a consequence of accelerated catabolism of TRL, these RNA-based therapies have the potential to significantly reduce plasma abundance of TRL-Aβ and protect the cerebral microvasculature. ANGPTL3 is a hepatically derived secretory protein that inhibits lipoprotein lipase activity. Vupanorsen, an antisense drug to ANGPTL3 mRNA markedly reduced triglycerides (~50%), apo CIII (~60%), remnant cholesterol (~38%), total cholesterol (~19%), non-HDL-cholesterol (~18%), but had more modest effects on plasma apo B (~9%) in subjects with T2DM and hepatic steatosis. The latter suggest, that whilst hydrolysis of TRL is markedly increased, the reduction in lipoprotein concentration per se is of less significance. Consistent with the latter, Katzmann et al. reported that in 195 subjects with stable CAD, that the apo C3 concentration in chylomicron free serum was associated with event free survival, but that the postprandial lipoprotein fraction (chylomicron) was not influenced by an oral fat challenge (55). Given that preclinical models suggest Aβ remains with the TRL-lipoprotein moiety throughout the metabolic cascade (22), a potential reduction in plasma Aβ homeostasis would be suggested for apo C3 and ANGPTL3 antisense therapies.

In summary, pharmacological agents that reduce plasma TRL levels may ‘correct’ plasma Aβ homeostasis and consequently reduce the risk of AD in diabetes.

### 6.1 Implications for diabetes-associated Alzheimer’s disease

A lipoprotein-Aβ/microvascular axis for onset and progression of AD would notionally provide a number of new therapeutic considerations. There is a substantial body of literature spanning many decades considering how dietary behaviour, exercise and pharmacotherapies modulate plasma lipid homeostasis, insulin sensitivity and risk for cardiovascular and metabolic disorders (56, 57). However, presently there is a paucity of information as to how said interventions impact on peripheral and central metabolism of amyloid.

Priority considerations to explore the hypothesis further are establishing robust analysis to study the kinetics and measure abundance of an exceedingly lipophilic protein that doesn’t lend itself readily to immunodetection methodologies. Preclinical studies in cell culture and animal models would be highly informative in considering how dietary fats, lipid-lowering, and insulin sensitising agents might modulate peripheral metabolism of lipoprotein-Aβ and impact on microvascular integrity.

Probucol an historic drug used clinically to lower cholesterol was shown to reduce TRL-Aβ secretion concomitant with preservation of BBB in high-fat fed diabetes models (11, 33, 48). The findings of the Probucol in Alzheimer’s trial in AD will be relevant to the hypothesis presented. In addition, newer generation pharmacotherapies such as mRNA silencing therapies targeted at apoC3 and ANGPTL3 that profoundly lower plasma triglyceride concentrations (58), may be worthy of priority consideration on the bases that the plasma abundance of TRL-Aβ may also be markedly decreased.

## 7 Limitations of hypothesis for risk of Alzheimer’s disease in diabetes

Whilst there is an accumulating body of scientific literature demonstrating an association between T2DM and AD, the aetiology is exceedingly complex. The general support for the role of vascular disturbances (such atherosclerosis) that may compromise tissue perfusion, microvascular disturbances associated with inflammation of the neurovascular unit, hyperoxidative stress and greater autophagic processes leading to accelerated neurodegeneration. Evidence of lipoprotein-Aβ capillary axis for AD in T2DM proposed in this review is based primarily on models of AD, or dietary fat-induced T2DM but remains to be proven in robust models of diabetes or clinically.

## Data availability statement

The original contributions presented in the study are included in the article/supplementary material. Further inquiries can be directed to the corresponding author.

## Author contributions

RT and JM developed the concept and wrote the manuscript. AS, EB, MM, DC, VL, and GW were involved in the manuscript writing and finalisation. AS and EB were responsible for the artwork. All authors contributed to the article and approved the submitted version.
